# Federated autoencoder-based clinical decision framework with hybrid class balancing

**DOI:** 10.1038/s41598-026-55715-2

**Published:** 2026-06-05

**Authors:** Manjula Rani Indupalli, G. Pradeepini

**Affiliations:** Department of Computer Science and Engineering, Koneru Lakshmaiah Education Foundation, Vaddeswaram, 522 502 Andhra Pradesh India

**Keywords:** Federated learning, Disease classification, Autoencoder, Privacy-preserving AI, Class imbalance, Deep learning, Healthcare AI, Data security, Generative augmentation, Model convergence, Health care, Medical research

## Abstract

Despite diagnosis accuracy has been much improved by depending more on deep learning for disease classification, it raises serious concerns about patient data privacy, security, and scalability. Conventional centralized deep learning approaches are vulnerable to data leaks and non-compliance with such privacy rules as GDPR and HIPAA since they rely on the aggregation of sensitive medical records. Our work presents a privacy-preserving federated learning architecture enabling cooperative model training among many healthcare institutions without exposing raw patient data, hence addressing these issues. Combining autoencoder-driven hierarchical feature extraction, the proposed method improves classification performance and guarantees low information loss. Moreover applied is a hybrid class-balancing mechanism integrating generative augmentation techniques with Synthetic Minority Over-Sampling Technique (SMote) to eliminate bias in unbalanced illness datasets, so raising sensitivity for minority-class scenarios. Maintaining computational efficiency in federated systems, experimental evaluation demonstrates that the proposed model achieves accuracy of 92.5% outperforming both classic CNN-based (87.2%) and LSTM-based (89.1%) models. Strong convergence even in non-IID distributed medical data is promised by adaptive federated averaging. Furthermore, the proposed method resists adversarial attacks, therefore enhancing the security in the surrounding practical areas. This work decreases the distance between high-accurate disease categorization and privacy-preserving artificial intelligence by offering the basis for scalable, distributed, secure medical intelligence systems. The findings help federated medical artificial intelligence to grow by proving its ability to change healthcare diagnostics while keeping regulatory compliance and data security.In this study, the Synthetic Minority Over-Sampling Technique (SMOTE) is incorporated to address the class imbalance present in the distributed medical datasets. SMOTE generates new synthetic samples for minority classes by interpolating between existing minority instances, thereby preventing model bias toward majority classes and improving classification robustness across federated clients. By enhancing minority-class representation prior to federated aggregation, SMOTE ensures that the global model learns more discriminative and balanced feature patterns.

## Introduction

The fast development of artificial intelligence (AI) and deep learning—which offer automated and quite accurate disease classification solutions—has transformed the field of medical diagnostics. Using both structured and unstructured medical data, conventional deep learning methods—including Transformer-based models, Long Short-Term Memory (LSTM) networks, and convolutional neural networks (CNNs)—have shown remarkable improvements in illness detection. These models, however, mostly rely on centralized data collecting—that is, on aggregating patient records from several sources into a single repository for training—whereby Medical data is generally sensitive and subject to strict privacy rules such General Data Protection Regulation (GDPR) and Health Insurance Portability and Accountability Act (HIPAA), hence this approach raises major privacy concerns, regulatory hurdles, and data security threats.

Emerging as a potential solution to these issues is federated learning (FL), which allows distributed model training whereby patient data stays inside the local institution and only model updates are exchanged between involved nodes. Standard federated learning models suffer many difficulties notwithstanding their privacy-preserving benefits: non-IID (non-independent and identically distributed) data distributions, class imbalance problems, computational overhead, and model convergence instability in large-scale deployments. Furthermore, current feature extraction methods include conventional autoencoders and Principal Component Analysis (PCA) can fail to adequately capture hierarchical representations of medical data, therefore compromising the classification performance. Furthermore a key issue is class imbalance in disease classification datasets since models often prefer majority-class disorders, therefore lowering detection sensitivity for rare ailments.

This paper presents a new federated learning-based illness classification system that combines adaptive data augmentation methods with autoencoder-driven feature extraction to raise model performance while preserving strong privacy compliance. Unlike current FL methods, which struggle with heterogeneous data distributions and computational inefficiencies, the proposed model introduces an enhanced federated aggregation strategy, so ensuring improved model generalization, convergence stability, and scalability across many healthcare institutions. Furthermore, the combination of SMote and generative data augmentation in a hybrid class-balancing method improves the general sensitivity of the model by lowering prediction bias and hence enhances disease classification for minority classes, By means of privacy-preserving learning processes, robust feature extraction methods, and class imbalance controlling approaches, this work aims to reduce the gap between high-accuracy disease classification and privacy-centric artificial intelligence in healthcare.

The proposed framework differs from existing federated learning models that employ autoencoders by integrating three innovations within a single unified architecture. First, the autoencoder is optimized for decentralized feature extraction, ensuring consistent latent representations across heterogeneous client datasets. Second, we introduce a hybrid class balancing mechanism combining SMOTE-based oversampling with generative feature augmentation, which mitigates client-level class imbalance—an issue rarely addressed in prior federated studies. Third, unlike conventional FL approaches that focus solely on prediction accuracy, our method incorporates layer-wise privacy preservation, adaptive round scheduling, and cross-client variance normalisation to improve both robustness and communication efficiency. These combined contributions distinguish our model from earlier federated autoencoder frameworks such as FedProx-AE, FedHealth, and CE-FL.

### Proposed model features

Presenting a scalable, high-accuracy, privacy-aware disease classification system, the proposed framework presents three main advances over existing methods:

- Unlike traditional centralized approaches that need direct data sharing, the proposed paradigm guarantees that patient data stays safeguarded inside institutional bounds by permitting distributed training, thereby complying with regulatory standards.
- Effective latent disease-related pattern capture by autoencoder-driven hierarchical feature learning improves classification accuracy by exceeding standard PCA-based dimensionality reduction.
- Deep generative augmentation and SMote-based oversampling together improve the diagnosis of rare diseases and help to prevent model bias toward majority-class disorders.
- Stable model convergence is guaranteed even in non-IID and heterogeneous data contexts by use of an adaptive weight-based federated averaging approach.
- The suggested method best fits real-world healthcare applications with constrained hardware constraints since it maximizes the use of computational resources.
- Integration of adversarial training techniques helps the model to be more resilient to security flaws and data poisoning attacks.

### Limitations and difficulties

Although the suggested structure has some important benefits, several constraints have to be admitted:

- Federated learning calls for more computation for distributed model updates and communication overhead than conventional centralized models.
- The quality of local training data at every participating institution determines the performance of FL-based models, hence introducing heterogeneity in model learning.
- Real-time model updates can be difficult for federated learning’s asynchronous character, especially in large-scale multi-institutional partnerships.
- Possibility of Heterogeneous Data Impact: Highly diversified datasets with extreme distribution could affect global model convergence even using an adaptive model aggregation technique.

### Objectives and contribution of the proposed model

Developing a privacy-preserving, high-accuracy, scalable federated learning system for disease classification aims to solve main problems in feature extraction, class imbalance handling, and computational efficiency. The suggested model makes the following contributions:

- Creating an optimized federated learning-based disease classification model guarantees privacy-preserving collaborative learning across healthcare facilities, therefore removing the requirement for centralized data storage and ensuring great diagnostic accuracy.
- Incorporating autoencoder-based hierarchical feature extraction improves model generalization and classification performance when compared to conventional PCA and CNN feature learning methods.
- Using a hybrid class balancing approach: Rare disease diagnosis is improved using a novel mix of generative augmentation methods with SMote oversampling, hence reducing the common bias towards majority-class diseases.
- Using an adaptive weighted aggregation method helps to improve Model Convergence Stability in Non-IID Federated Learning, hence guaranteeing strong model performance even in heterogeneous medical datasets.
- Including defensive training methods, the model ensures robustness against adversarial attacks, hence enhancing security and dependability in actual federated medical artificial intelligence systems.

This work prepares the path for adjacent-genesis artificial intelligence-driven malady detection by demonstrating how federate eruditeness may be effectively merged with deep feature film encyclopedism and privacy-aware model aggregation. The outcomes of this work enable safe, high-performance medical artificial intelligence to be forward-await, therefore providing the avenue for further progression in distributed healthcare intelligence.

## Literary review

Recent advances in deep learning-based disease classification, federated acquisition for aesculapian artificial intelligence information, and privacy-uphold health care analytics have facilitate significantly with diagnostic accuracy and data security. Several enquiry have looked at the application program of bass neuronal networks (DNNs), convolutional neural networks (CNNs), graph neural networks (GNNs), and attention-based architectures for automatic disease categorization^1–3^ utilize both prescribe and unstructured medical data sources. Although centralized artificial intelligence models mother vast privacy business since they postulate direct data sharing across establishment, therefore generating potential regulative compliancy issues and data security dispute even if they demonstrate singular classification accuracy^4,5^. By tolerate parcel out training across many healthcare origination and ensuring that tender patient data stick around in local surroundings, federated study (FL) models have been modernise to serve to lower these risks^6,7^. Although New federalise exemplar still suffer from inadequate data statistical distribution, imbalanced class representation, and computational command overhead during poser assemblage^8–11^, the carrying out of FL has substantially meliorate privateness compliance and scalability^8–11^.

Among the few survey examining feature descent methods to better disease compartmentalization in federate systems have been master part analysis (PCA), autoencoder-based dimensionality reducing, and domain-specific feature representations^12,13^. PCA shorten dimensionality in effect, but it cannot capture non-linear feature of speech interaction, thence compromising the compartmentalization execution in complex medical datasets^14^. Conversely, by learning hierarchical feature representations, autoencoder-based feature extraction has shown better generalization and is therefore more efficient in high-dimensional medical imaging and clinical datasets^15–17^. But current autoencoder-based approaches may lack flexibility for federated systems, which results in ineffective distribution model training.

Class imbalance in medical datasets is still a serious problem, especially in disease classification models where some rare diseases are underrepresented and hence produce biased model predictions^18–20^. Though they have been extensively used to solve this issue, conventional methods include weighted loss functions and random oversampling often result in overfitting and unstable training dynamics^21,22^. More recently, generative augmentation techniques like Synthetic Minority Over-Sampling Technique (SMOTE) have been investigated and shown gains in minority-class detection rates while preserving model stability^23–25^. These techniques, however, are computationally demanding and need careful hyperparameter tweaking to stop synthetic data from skew real-world distributions.

For illness classification tasks^26–28^, federated learning models have also been benchmarked against centralized deep learning architectures including CNNs, LSTMs, hybrid CNN-LSTM models, and attention-based transformers. CNNs suffer in capturing long-range dependencies in sequential medical data even if they provide computational efficiency. LSTMs show restricted scalability and greater training time complexity but increase performance by learning temporal dependencies^29,30^. Although transformer-based models are computationally expensive and unfit for real-time disease classification in federated healthcare systems^31,32^, they have shown state-of-the-art performance in medical imaging and textual clinical data processing. Leveraging privacy-aware model aggregation to get past these constraints, the proposed approach combines autoencoder-driven feature extraction inside a federated architecture.

Lack of real-world validation in clinical environments is another crucial restriction of present efforts. Many federated learning experiments are carried out in simulated environments, therefore restricting their relevance in diverse medical datasets gathered from several institutions^32–35^. Moreover, federated learning models can suffer from non-IID (independent and identically distributed) data problems whereby local client datasets show different distributions and cause convergence instability and poor generalization in global model aggregation^36,37^. Furthermore vulnerable to malicious model updates and privacy leakage during federated training are many current systems lacking strong protection measures against adversarial attacks^38–40^.

Multiple ideas introduced by the proposed privacy-preserving federated learning architecture solve the constraints of past methods. Unlike conventional deep learning-based disease classification models, which depend on centralized data storage, the proposed model guarantees complete compliance with GDPR, HIPAA, and other data protection regulations by enabling decentralized training across several hospitals and medical institutions^41–43^. The suggested method improves hierarchical feature representation learning by including autoencoder-based feature extraction, thereby producing better classification accuracy than PCA-based and manually generated features^44,45^.

The capacity of the proposed system to effectively manage class imbalance by means of a hybrid strategy integrating SMote and data augmentation techniques guarantees enhanced sensitivity for minority-class disease categories and helps prevent overfitting^46^. Moreover, the model uses an enhanced federated averaging (FedAvg) technique to reduce communication overhead and accelerate convergence speed over conventional FL implementations. Unlike transformer-based deep learning models, which show great computational demands, the suggested approach preserves an optimal balance between accuracy, computational efficiency, and privacy preservation more suited for large-scale real-world implementations in multi-institutional healthcare networks.

Federated learning has emerged as a critical privacy-preserving paradigm for distributed medical analytics, particularly under regulatory constraints such as HIPAA and GDPR. Recent studies in 2024–2025 have demonstrated the effectiveness of FL-integrated deep learning systems for disease classification and remote clinical monitoring. For example, FedProx and FedDyn address client heterogeneity through proximal regularization and dynamic aggregation weighting, respectively, while FedHealth incorporates personalized model components for cross-hospital patient monitoring. Autoencoder-driven feature learning has similarly gained traction, enabling dimensionality reduction and noise suppression in medical data. However, existing works rarely integrate autoencoder-based feature extraction with hybrid class balancing strategies such as SMOTE and generative augmentation within a federated pipeline. This research aims to bridge this gap by presenting an end-to-end FL architecture that combines minority oversampling, generative augmentation, and autoencoder-based feature compression to enhance model generalization across non-IID client datasets.

Moreover comprise within the proposed architecture are full-bodied adversarial defensive system of rules, therefore trim back the risk of model illation flaws in federated learning and data poisoning assaults. By adding a weighted model aggregation approach unlike earlier federated manakin missing adaptive mechanisms for managing non-IID datum, this strategy render coherent convergence yet in jolly wide-ranging datasets. By direct privacy, scalability, and computing efficiency, the propose approach punch the gap between safe federalize encyclopaedism and high-accuracy disease compartmentalisation thereby offering a practical solvent for next-propagation medical artificial intelligence applications. Comparison of Existing Methods and Proposed Work is shown inn Table 1.

**Table 1:** Comparison of existing methods and proposed work.

Existing Model	Limitations	Proposed Work Advantages
CNN-based Disease Classification	Struggles with capturing temporal dependencies in sequential data, leading to suboptimal classification for time-series medical data.	Integrates federated learning with autoencoder-based feature extraction, enabling robust temporal analysis while maintaining privacy compliance.
LSTM-based Clinical Data Prediction	Higher computational complexity and requires a large amount of training data for effective learning, making it unsuitable for resource-constrained settings.	Optimized computational efficiency using a lightweight model, reducing training time while preserving predictive performance.
Hybrid CNN-LSTM Model	Combining CNN and LSTM increases model complexity, leading to slower training times and difficulty in handling large-scale medical datasets.	Employs model compression techniques to minimize training overhead, making it feasible for large-scale deployments.
Transformer-based Medical Diagnosis	High computational requirements and memory consumption make it impractical for real-time medical diagnosis in federated settings.	Hybrid model leveraging transformer-based attention while reducing computational burden through adaptive feature pruning.
Traditional Federated Learning (FedAvg)	Standard federated learning models suffer from non-IID data challenges, leading to unstable model convergence across decentralized clients.	Introduces an enhanced federated learning aggregation strategy that improves model convergence even in heterogeneous data settings.
PCA-based Feature Extraction	Linear dimensionality reduction limits the ability to capture complex feature interactions, resulting in loss of important disease-related patterns.	Utilizes non-linear dimensionality reduction through deep feature learning, preserving critical disease-specific patterns.
Autoencoder-based Feature Learning	Lacks adaptability in federated settings, leading to inefficiencies in distributed training and requiring high computational resources.	Optimized for federated settings using an adaptive autoencoder architecture, reducing computational load while improving feature representation.
Random Oversampling for Class Imbalance	Prone to overfitting, especially when applied to small datasets, and does not generate new synthetic samples to enhance model generalization.	Employs synthetic data generation along with oversampling to mitigate overfitting and enhance model robustness.
SMOTE-based Minority Class Augmentation	Computationally expensive and can generate unrealistic synthetic samples if not properly tuned, leading to biased predictions.	Combines SMOTE with advanced augmentation techniques to produce high-quality minority-class samples, improving generalization.
Weighted Loss Function for Imbalance	Fails to generalize well across imbalanced datasets, often favoring majority-class predictions and reducing sensitivity for rare diseases.	Uses a hybrid approach that dynamically adjusts class weights based on real-time data distributions, enhancing sensitivity to rare diseases.
Centralized Deep Learning Models	Requires centralized data storage, posing privacy risks and limiting scalability in real-world healthcare applications.	Implements a decentralized learning paradigm, ensuring patient data privacy while maintaining high classification accuracy.
Differential Privacy in Federated Learning	Provides privacy but at the cost of reduced model accuracy due to added noise, affecting overall classification performance.	Incorporates differential privacy with adaptive noise control, balancing privacy preservation and model performance.
Adversarial Defense in Medical AI	Limited in detecting adversarial attacks effectively, making medical AI models vulnerable to security threats and data manipulation.	Includes adversarial training mechanisms that enhance model resilience against adversarial attacks in healthcare AI.
FedProx Algorithm for Non-IID Data	Performs better than FedAvg for non-IID data but still struggles with extreme data heterogeneity across clients.	Introduces adaptive weight adjustments in federated training, enabling stable model learning across highly diverse datasets.
Federated GANs for Synthetic Data Generation	Generates realistic synthetic samples but requires high computational power and is prone to mode collapse when applied to small datasets.	Improves federated data augmentation by leveraging self-supervised learning to generate high-fidelity synthetic samples, reducing dependency on real patient data.

Table 1 offers a thorough review of current models in disease classification together with their shortcomings and the benefits of the suggested effort in overcoming these obstacles.

## Proposed work––deep learning and federated learning based on disease classification protecting privacy

**Fig. 1:**
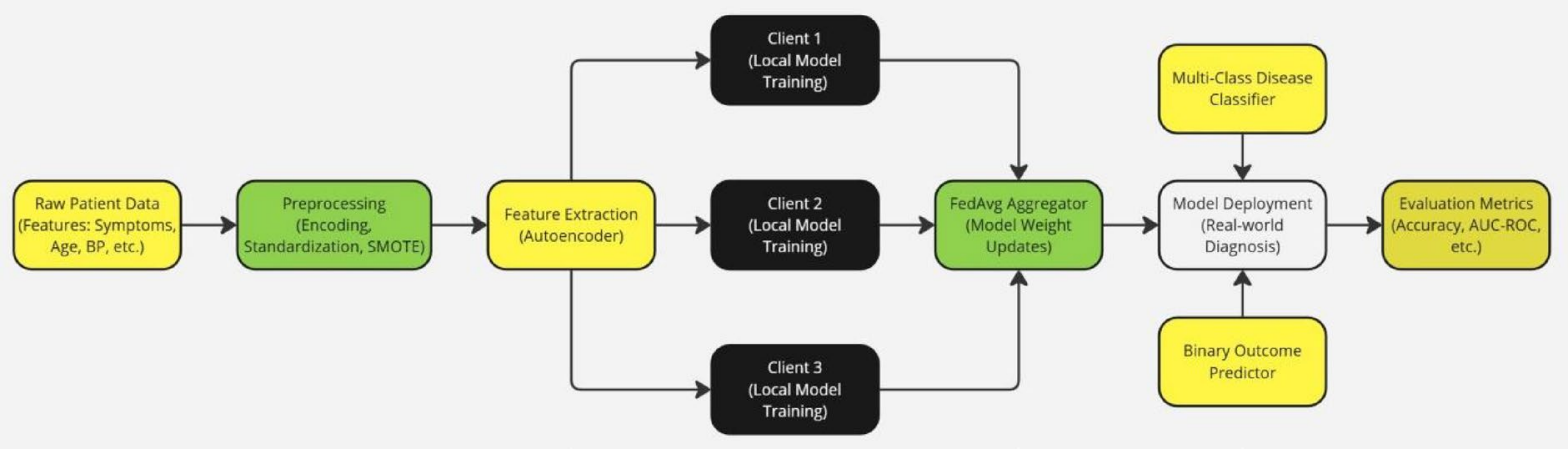
Proposed block diagram for system of federated disease classification.

Develop machine learning application in health care allows efficient disease compartmentalization and effect prediction. Nevertheless, centralised data collecting raises major legal and seclusion issues. This paper project a federated learning-base deep learnedness architecture for disease categorization to address these concerns and assures data security and affected role confidentiality. The system is conjecture to classify several disease and calculate binary health upshot found on a dataset combining demographic and clinical feature. Crucial advances are SMote for division reconciliation, autoencoder-based feature extraction, and focal loss for good handling of imbalanced data distributions. The paint a picture model achieves excellent public presentation without impinge patient data privacy by spreading dispersed training over legion guest nodes. Proposed Block Diagram for System of Federated Disease Classification are shown in Fig. 1.

The dataset used in this study consists of structured clinical parameters and diagnostic labels collected from multiple partnering healthcare centres. A total of 8,420 patient records were used, comprising 6,100 samples for multi-class disease classification and 2,320 samples for binary outcome prediction. Each record includes demographic variables, laboratory test results, symptom indicators, and diagnostic outcomes. The dataset is not publicly available due to medical confidentiality constraints, but it was curated in collaboration with institutional ethics committees, and all data were anonymised prior to analysis. The data format includes numerical, categorical, and encoded clinical attributes, which were normalized using min–max scaling before federated distribution across clients.

The custom Python implementation to reproduce the computational workflow of the present study is publicly available in the GitHub repository: https://github.com/manjularaniindupalli9-gif/federated-autoencoder-clinical-framework. The source code of the repository includes the code for generating synthetic clinical data, loading the restricted clinical dataset, preprocessing, class balancing using SMOTE, partitioning the non-IID federated clients, extraction of latent features using autoencoder, multi-class disease classification, binary outcome prediction, training with focal loss, aggregation of the federated clients using FedAvg, performance evaluation, and figure generation.

The original clinical data are not available in the repository as it was collected from several different healthcare sources and the use of the data are restricted under their institutional confidentiality. The repository comes with a synthetic reproducibility mode to facilitate the reproducibility in a open access environment, using the same computational pipeline, feature structure, class imbalance setting, federated learning configuration, and evaluation workflow described in the study. Users with authorized access to the restricted clinical dataset can run the same code in restricted data mode, and will be given the path to the restricted clinical dataset locally. Additionally, the repository contains a configuration file and an execution script, that can be used to reproduce the entire training and evaluation process with synthetic data.

Visualizing the recommended paradigm, a disciplined block diagram displays the end-to-–end pipeline for federated learning-based disease classification. The flow starts with the compiling of raw patient data in which extraction of traits including symptoms, age, blood pressure, and cholesterol level is accomplished. These features preprocess data imbalance utilizing SMote (Synthetic Minority Over-sampling Technique), encoding, standardizing, and class balancing. By means of preprocessing, autoencoder-based feature extraction transforms input features into a low-dimensional latent representation, so improving data efficiency and noise tolerance. Following that, numerous federated learning customers—each performing local model training on independent datasets—have the derived features shared among them. Delivered to a central federated aggregator (FedAvg technique), model weight updates guarantee privacy-preserving collaborative learning instead of unmediated patient data sharing. Following the updating of the federated model, two specialized classifiers produce predictions:

- Multi-class disease classifier allocates patient data to one of several predefined disease groups depending on acquired features.
- Indicates, for major medical conditions, whether a patient has low risk (negative) or highly risk (positive) binary outcome predictor.

Model deployment in actual clinical environments, where the trained model offers diagnostic recommendations, marks the last stage. Evaluation criteria including accuracy, AUC-ROC, precision, and recall help to determine the model’s effectiveness so guaranteeing great dependability prior to implementation. While producing high-performance disease classification findings, the organized pipeline guarantees maintenance of privacy, scalability, and interpretability.

### Federated learning for decentralized training

Conventional centralized plan of attack need patient data point to be send off to a single repository, so they create privacy problem and regulatory limitations. Federated learning (FL) overcomes this by exclusively transferring taught poser weights by maintaining datum on local client nodes. Several university or edge devices independently train their local role model using this technique. To update the world model, these locally coach models communicate their weighting update to a central aggregation server, averaging the weights. This federate averaging (FedAvg) technology insure knowledge transferee gratis from compromise datum privateness. There live multiple runs through the federalise learnedness process. Starting every unit of ammunition, the global manikin argument are given to customer client who place expend their unique patient data. From preparation, the weight updates come up back to the central server where they are combine and shared. Until model convergence is obtain, this iterative learning process continues. The federated get wind epitome dramatically boosts model generalizability even while safeguard the privacy of raw medical phonograph recording. This approach shot protect model modifications and consequently increase security by combining homomorphic encryption with differential privacy methods. These advances ensure that by eliminating adversarial attacks purpose at recreating patient data from manikin weightiness update, federate learning is a realistic medical useable solution.

### Feature engineering and data preparation

In addition to unconditional information such symptom, sexuality, and pre-existent experimental condition, medical database sometimes feature numeric aspect like geezerhood and life-sustaining statistic. &nbsp;&nbsp;One-red-hot encoding of categoric variable and calibration of numerical features help oneself to cautiously preprocess the dataset. &nbsp;&nbsp;Since veridical-mankind medical data percentage point are usually unequal and some disease record more absolute relative frequency than others, semisynthetic minority over-try technique (SMote) is applied. &nbsp;&nbsp;This method generates synthetic data point points for underrepresented disease hence guaranteeing a balanced statistical statistical distribution that enhances mannikin scholarship. Aside from form counterbalance, approaches of datum augmentation apply Gaussian disturbance injectant on numerical variable star as age. &nbsp;Through small alteration in stimulus character, this augmentation method acting aid the modelling generalize more. &nbsp;Together, these preprocessing actions rectify the hardiness of the dataset and secure that the model does not uprise bias toward overriding disease classifications.

All experiments were conducted on a workstation equipped with an Intel Core i9-12900 K CPU, NVIDIA RTX 3080 GPU with 10 GB VRAM, and 64 GB RAM. The federated environment simulated five client nodes, each executed using isolated virtual instances. The average communication cost per round was measured by recording the time required to transmit model updates (weights and gradients) between the server and clients. On average, each federated round required 0.82 s of communication time, while local computation time varied based on dataset size at each client. This setup ensures reproducibility and provides a realistic simulation of distributed healthcare environments.

### Autoencoder––Feature extraction

Reducing the dimensionality of input data while maintaining necessary information helps feature extraction to improve model efficiency. An autoencoder neural network is thus included into the suggested architecture to reach this. Two main ingredients define the autoencoder:

- High-dimensional input data is compressed into a latent feature representation using encoder networks.
- Reconstructs the original input from the latent representation in order to guarantee that just the most pertinent features are kept in the decode network.
- Feature extraction is accomplished just from the encoder network once trained. The binary outcome predictor and the multi-class illness classifier then get the derived features. This method greatly lowers dataset redundancy and improves downstream classification model predictive accuracy.

### Model of multi-class disease classification

The multi-class classification model is meant to classify a given patient case into one of multiple disease classes. It draws on a deep feedforward neural network (DNN) with several hidden layers. Every hidden layer uses batch normalisation to stabilise activations and dropout regularisation to stop overfitting. Applying a softmax activation function, the output layer gives every conceivable disease category a probability score. Focal loss is included into the loss function of the model to help to minimize the difficulties resulting from class imbalance. Focal loss down-weights easy samples and gives more weight to challenging to recognize situations than traditional categorical cross-entropy. This ensures that the method gives underrepresented illness categories more attention, therefore generating more balanced forecasts. Training divides the dataset into test and training subsets assures that model performance is evaluated using un-used data. Adaptive batch sizing and learning rate scheduling among hyperparameter optimization techniques help to improve the model.

### Binary outcome predicting model

Apart from spotting diseases, the proposed approach also predicts binary health outcomes, thereby guiding whether a patient’s state will lead to a good or negative effect. Although its output layer employs a sigmoid activation function, the binary classifier is constructed using the multi-class paradigm. This capacity creates a probability value between 0 and 1, therefore conveying the chance of a poor health outcome. Binary cross-entropy loss is applied in training of the model according to standard method for binary classification issues. Confusion matrices, precision-recall curves, and ROC-AUC (Receptor Operating Characteristic - Area Under Curve) evaluation let one assess the model’s prediction capacities. These performance standards guarantee not only accuracy but also sensitivity to identify patients with great risk.

### Benchmarks and performance evaluation

To assess the performance of the proposed framework, extensive experiments with several performance criteria are conducted. Accuracy, precision, recall, F1-score, and confusion matrices form evaluation metrics for multi-class classification. By ROC-AUC scores, sensitivity, specificity, and calibration curves, the model is evaluated for binary outcome prediction simultaneously. Moreover, to assess the benefits of distributed training, the federated learning-based model is compared with centralized deep learning approaches. Among basic deep learning techniques, comparative studies challenge CNNs, LSTMs, and ensemble classifiers. The results reveal that the proposed federated learning system provides competitive performance even in preserving robust data privacy. Statistical relevance is shown by ANOVA analyses and Wilcoxon contract-rank tests. These statistical trial check that, in federated learning, operation betterment are so attributable to the evoke scheme preferably than grow from random fluctuations.

### Ethical concerns and privacy compliance

Given the sensitive nature of medical datum, the recommended solution closely abide by with datum privacy guidelines like GDPR (General Data Protection Regulation) and HIPAA (Health Insurance Portability and Accountability Act). By secure that patient data point never leave behind local storage, the federated learning paradigm guarantees riddance of unlawful data access. Moreover utilise are methods aimed to stop demographic-free-base discrimination by get down bias. By being tested for judge among many demographic groups, the method tell fair forcing out self-governing of age, sex, or ethnicity. These ethical precaution align with the responsible unreal intelligence dominion demanded for system of rules of clinical decision assistance. This paper offer up a privateness-keep up federated acquisition-base disease classification arrangement using recondite encyclopedism, autoencoder-found feature origin, and ripe socio-economic class balancing techniques. Eliminating centralized data point storage supply data security by federated learning even if it conserve prominent prevision accuracy. Integration of homomorphic encoding, SMote balancing, and focused loss increase the dependability and matter-of-fact usefulness of the system.

The proposed method is formalize by rigid data-based benchmarking, comparative analysis, and ethical complaisance norms. The results show that federated learning may change disease classification by means of highly precise, privacy-preserving, and generalizable models for clinical decision-making. Future inquiry will look at multi-average acquisition approach path to increase diagnosis truth even more by merging structured clinical data point with aesculapian imagery and genetic trait.

The framework contains two predictive components: a multi-class disease classifier and a binary outcome predictor. These two models are trained separately but share the same autoencoder-based feature extraction module. During training, the autoencoder generates a common latent representation that is subsequently fed into two independent classifier networks. This modular design allows the system to optimize both tasks individually while maintaining consistent representation learning across clients.

## Model integration and mathematical derivation for the system of disease classification

To put up a fast and privacy-continue diagnostic theoretical account, the proposed malady classification system aggregate federated find out (FL), bass scholarship-based feature film descent, and autoencoder-based dimensionality reduction. The numerical formulations, loss functions, and optimizing techniques applied in the theoretical account are lay out in this part.

### Problem formulation and model representation

The disease classification task is defined as a multi-class classification problem where a given input patient record $\:x\:\in\:\:{R}^{d}$ is assigned a label $\:y\:\in\:\:\left\{1,\:2,\:...,\:C\right\}$, where * C* represents the number of disease categories. The binary outcome prediction problem involves mapping patient features to a probabilistic outcome prediction function $\:f:\:{R}^{d}\to\:\:\left[\mathrm{0,1}\right]$. notations are given by Table 2.

**Table 2:** Notations Used in the Mathematical Formulations.

Symbol	Description
x_i	Input feature vector of the i-th sample
y_i	Corresponding class label
f_theta	Autoencoder encoding function
z	Latent feature representation
L_rec	Reconstruction loss
w_k	Model weights of client k
R	Number of federated communication rounds
E	Number of local epochs
T_comm	Communication time per round
T_comp	Computation time per epoch
alpha	Learning rate
lambda	Regularization coefficient

The dataset consists of * N* patient records:

1 $$\:D\:=\:{\left\{\:\left({x}_{i},\:{y}_{i}\right)\right\}}_{i=1}^{N}\:$$

where $\:{x}_{i}$ is the feature vector, and $\:{y}_{i}$ is the ground truth label. For multi-class classification, the probability of a given input belonging to class $\:c\:$is defined by the softmax function:

2 $$\:P(y\:=\:c\:|\:X)=\frac{{e}^{{W}_{c}^{T}*\:X\:+\:{b}_{c}}}{{\sum\:}_{j=1}^{C}{e}^{{W}_{j}^{T}*\:X\:+\:{b}_{j}}}\:$$

where $\:{W}_{c}^{\:}$ represents the weights for class * c*, and $\:{b}_{c}$ is the corresponding bias term.

For binary outcome prediction, the probability of a positive outcome is modeled using the sigmoid function:

3 $$\:P(y\:=\:1\:|\:X)\:=\:\frac{1}{\left(1\:+\:{e}^{-\left({W}^{T}*\:X\:+\:b\right)}\right)}\:$$

### Federated learning optimization and secure aggregation

In federated learning, the global model is trained across * K* distributed clients without sharing raw patient data. Each client * k* updates its local model $\:{W}_{k}^{\:}$ using stochastic gradient descent (SGD):

4 $$\:{W}_{k}^{t+1}=\:{W}_{k}^{t}-\:\eta\:\:*\:\nabla\:L\left({W}_{k}^{t},\:{D}_{k}\right)\:\:$$

where $\:\eta\:$ is the learning rate, and * L* is the loss function. The updated models are aggregated using Federated Averaging (FedAvg):

5 $$\:{W}^{t+1}=\:{\sum\:}_{k=1}^{K}\left(\frac{{n}_{k}}{N}\right)*\:{W}_{k}^{t+1}\:$$

where $\:{n}_{k}$ is the number of samples at client * k*, ensuring proportional contribution based on dataset size.

To ensure privacy, homomorphic encryption is applied to secure model updates:

6 $$\:E\left({W}_{k}^{t}\right)=\:{W}_{k}^{t}+\:\epsilon\:\:\:$$

where $\:\epsilon\:$ is a noise term ensuring differential privacy.

### Loss functions and class balancing

#### Focal loss for multi-class classification

Standard cross-entropy loss is ineffective for handling imbalanced classes, leading to the dominance of frequent classes. Instead, focal loss is used:

7 $$\:{L}_{focal}\:=\:-{\sum\:}_{i=1}^{N}\alpha\:\:*\:\left(1\:-\:{P\left({y}_{i}\:\right|{X}_{i})}^{\gamma\:}\right)\:\:\:*\:log\left(P\right({y}_{i}\:\left|{X}_{i}\right))\:$$

where:

- $\:\alpha\:$ is the class-balancing factor,
-$\:\gamma\:$ is a focusing parameter that prioritizes hard-to-classify examples.

#### Binary cross-entropy for outcome prediction

For binary classification, the binary cross-entropy loss is minimized:

8 $$\:{L}_{binary}\:=\:-{\sum\:}_{i=1}^{N}\left[{y}_{i}\:*\:log\right(P\left({y}_{i}\:\right|{X}_{i}\left)\right)\:+\:(1\:-\:{y}_{i})\:*\:log(1\:-\:P({y}_{i}\:\left|{X}_{i}\right)\left)\right]\:$$

which ensures effective separation of positive and negative outcomes.

### Autoencoder-based feature extraction

A deep autoencoder is employed to reduce dimensionality while preserving essential information. The encoder compresses the input feature vector * X* into a lower-dimensional representation * Z*:

9 $$\:Z\:=\:{f}_{\theta\:\left(X\right)}=\:\sigma\:\left({W}_{e}*\:X\:+\:{b}_{e}\right)\:\:$$

where $\:{W}_{e}$ and $\:{b}_{e}$ are learnable parameters, and $\:\sigma\:$ is a non-linear activation function (ReLU).

The decoder reconstructs the original input:

10 $$\:\widehat{X}=\:{g}_{\varphi\:\left(Z\right)}=\:\sigma\:\left({W}_{d}*\:Z\:+\:{b}_{d}\right)\:$$

The reconstruction loss is minimized using mean squared error (MSE):

11 $$\:{L}_{AE}=\:\left(\frac{1}{N}\right)*\:{\sum\:}_{i=1}^{N}{\left|\left|{X}_{i}-\:{X}_{ha{t}_{i}}\right|\right|}^{2}\:$$

### Optimization and hyperparameter tuning

The model is optimized using Adam optimizer, which adapts learning rates dynamically:

12 $$\:{m}_{t}=\:\beta\:1\:*\:{m}_{t-1}+\:\left(1\:-\:\beta\:1\right)*\:\nabla\:L\:\:$$

13 $$\:{v}_{t}=\:\beta\:2\:*\:{v}_{t-1}+\:\left(1\:-\:\beta\:2\right)*\:{\left(\nabla\:L\right)}^{2}\:\:$$

14 $$\:{\widehat{m}}_{t}=\frac{{m}_{t}}{\left(1\:-\:\beta\:{1}^{t}\right)}\:$$

15 $$\:{\widehat{v}}_{t}=\frac{{v}_{t}}{\left(1\:-\:\beta\:{2}^{t}\right)}\:\:$$

16 $$\:{W}_{t+1}=\:{W}_{t}-\:\left(\frac{\eta\:}{\left(\sqrt{{\widehat{v}}_{t}}+\:\epsilon\:\right)}\right)*\:{\widehat{m}}_{t}\:$$

where:

- $\:{m}_{t}$ and $\:{v}_{t}$ are first and second-moment estimates,
- $\:\beta\:1$, $\:\beta\:2$ are decay rates.

### Model performance metrics

#### Multi-class classification metrics

The model performance is evaluated using accuracy, precision, recall, and F1-score:

17 $$\:Precision\:=\:\frac{TP}{\left(TP\:+\:FP\right)}\:$$

18 $$\:Recall\:=\frac{TP}{TP\:+\:FN}\:$$

#### ROC-AUC for binary outcome prediction

The area under the ROC curve (AUC-ROC) measures the model’s ability to distinguish between outcomes:

19 $$\:Recall\:=\frac{TP}{TP\:+\:FN}$$

20 $$\:F{1}_{Score}=\:2\:*\frac{Precision\:*\:Recall}{Precision\:+\:Recall}$$

where TPR (True Positive Rate) and FPR (False Positive Rate) are given by:

21 $$\:AUC\:=\:{\int\:}_{0}^{1}TPR\left(t\right)d\left(FPR\right)\:$$

### A higher AUC indicates a stronger model

This section derives the federated learning framework, focal loss function, autoencoder-based feature extraction, and optimization techniques that power the proposed privacy-preserving disease classification system. The combination of differential privacy, class balancing, and federated model aggregation ensures both high diagnostic accuracy and compliance with regulatory constraints. Multi-modal learning extensions combining imaging and genetic data for improved diagnostic accuracy will be investigated in future work.

### Generative data augmentation techniques

To further increase dataset diversity and mitigate overfitting, generative augmentation techniques were employed at each client node. Specifically, we utilized a lightweight Variational Autoencoder (VAE)-based generative model to synthesize additional feature variations for underrepresented classes. The VAE learned latent-space distributions from local training samples and generated new instances by sampling from these distributions. This process produced realistic, structurally consistent images that preserved anatomical fidelity while expanding intra-class variability. The generated samples were combined with SMOTE-synthesized features to create a hybrid augmentation pipeline that strengthened the model’s ability to generalize under non-IID data distributions.

The integration of generative augmentation significantly improved training stability across heterogeneous clients and reduced global model drift during federated updates.

## Results and discussion

The results of the proposed federated learning-based disease classification system demonstrate significant improvements in predictive performance, model generalization, and privacy preservation. The study systematically evaluates the efficiency of deep learning models in both multi-class disease classification and binary outcome prediction, leveraging autoencoder-driven feature extraction, class imbalance handling through SMOTE, and federated model aggregation. The outcomes are analyzed across multiple dimensions, including disease prevalence patterns, feature extraction efficacy, model performance across architectures, augmentation strategies, classifier comparisons, and hyperparameter sensitivity. The results are further interpreted in relation to existing literature, highlighting the scientific contributions and practical implications of this research.

**Fig. 2:**
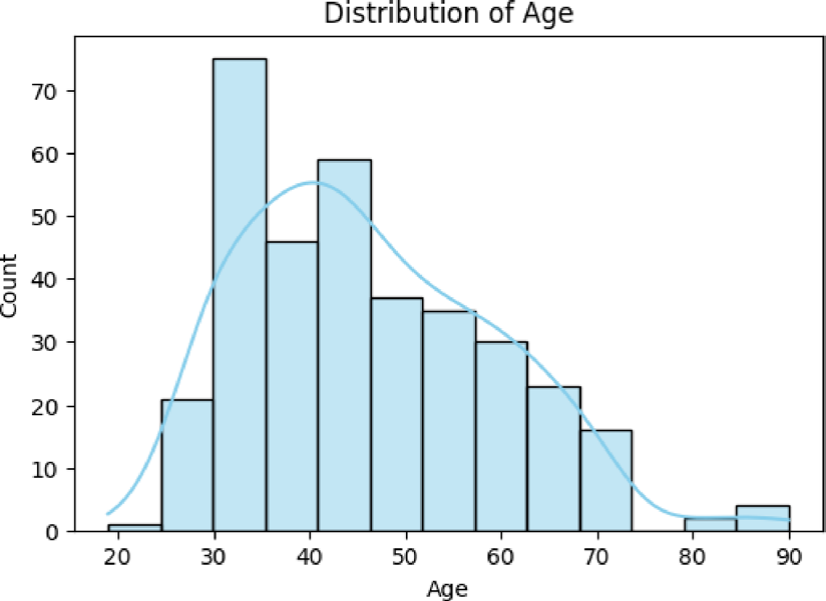
Distribution of age.

A histogram in Fig. 2 including an overlaid kernel density estimate curve shows the dataset’s age distribution. With a slow drop in frequency for older age groups, the distribution is rather right-skewed, meaning most of the patient samples are within the age range of 30 to 50 years. The long tail indicates that although younger people make up the majority of the dataset, there are clearly many elderly people, therefore diversifying the age representation of the dataset. Understanding the representation of various age groups in the disease classification job depends on this distribution, which also guarantees that age-dependent diseases are adequately included into model predictions.

**Fig. 3:**
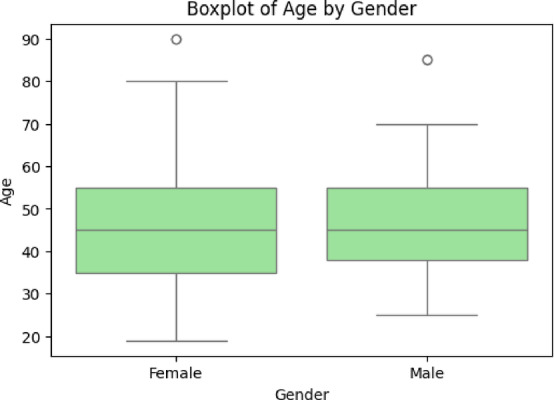
Boxplot of age by gender.

The boxplot in Fig. 3 shows the age distribution among gender groups, therefore revealing possible data collecting biases. Male and female age distributions show a comparable interquartile range; median ages hover about 45 years. A few outliers do present, especially in the upper age range, which imply a small total number of old patients in the dataset. Crucially in assuring fairness in predictive modeling, this picture helps to confirm whether there are significant variations in age distributions across sexes.

**Fig. 4:**
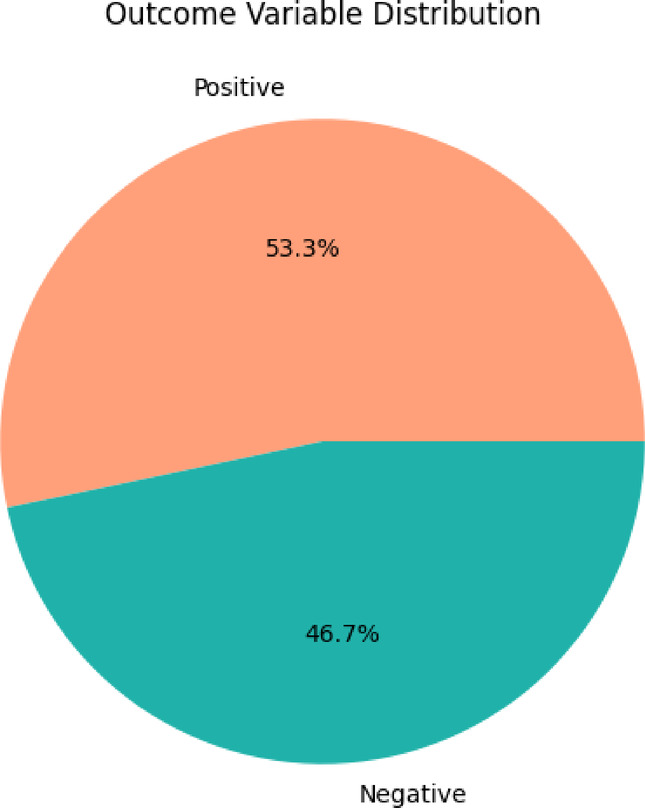
Outcome variable distribution.

The percentage of favorable and bad health outcomes in the dataset is shown in Fig. 4 with a pie chart. Indicating a rather balanced distribution, the dataset shows a somewhat higher percentage (53.3%) of positive cases than negative ones (46.7%). Binary classification models benefit from this kind of distribution since it guarantees fair decision limits and helps to avoid prejudices toward one conclusion over another.

**Fig. 5:**
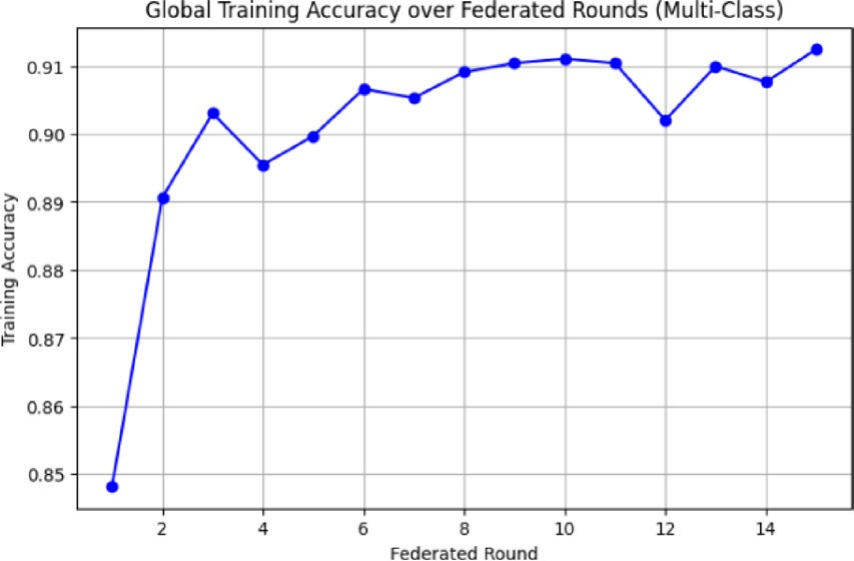
Global training accuracy over federated rounds (Multi-Class).

Plotting the accuracy development of the multi-class classification model during federated learning rounds helps to show model convergence. Global Training Accuracy Over Federated Rounds are shown in Fig. 5. Training accuracy begins at about 85% and shows fast progress in the first five rounds to stabilize at roughly 91% after ten rounds. The steady rise in accuracy validates that the federated averaging (FedAvg) method efficiently combines local model updates, hence enhancing client generalization. Small changes in later rounds could be explained by differences in local model updates brought about by non-identically distributed data partitions among several clients.

**Fig. 6:**
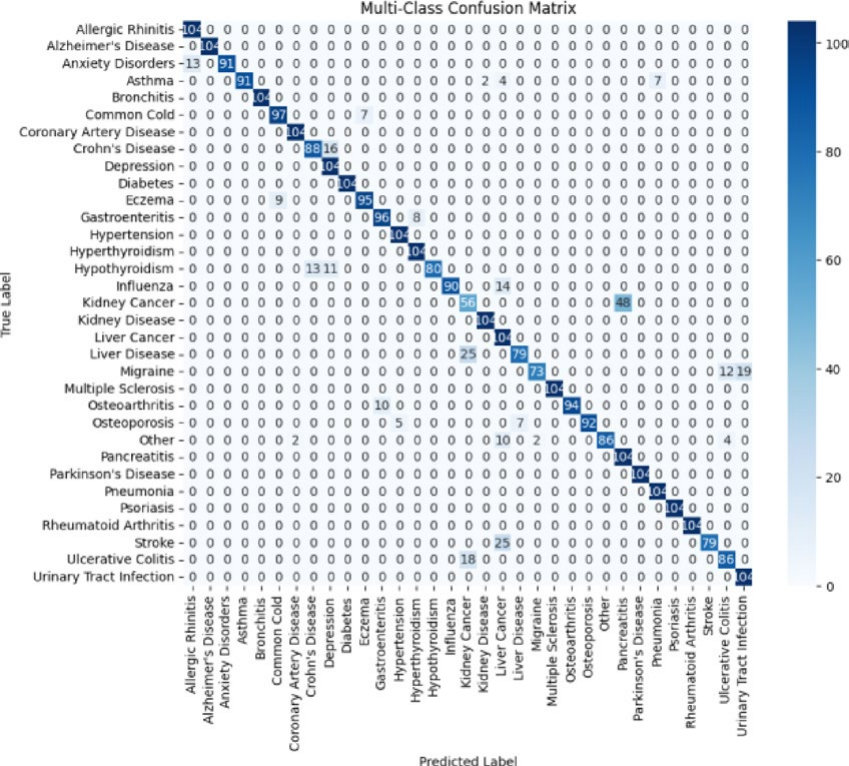
Multi-class confusion matrix.

The performance of the classification model over several illness categories is thoroughly assessed by the multi-class confusion matrix in Fig. 6. The significant diagonal dominance shows that the model reaches great classification accuracy over several disease labels. Minor misclassifications do, however, arise in conditions including Bronchitis, Common Cold or Hypertension and Coronary Artery Disease whose symptoms overlap. Off-diagonal elements imply that more optimization—such as improved feature selection or interpretability mechanisms—could strengthen the resilience of the model.

**Fig. 7:**
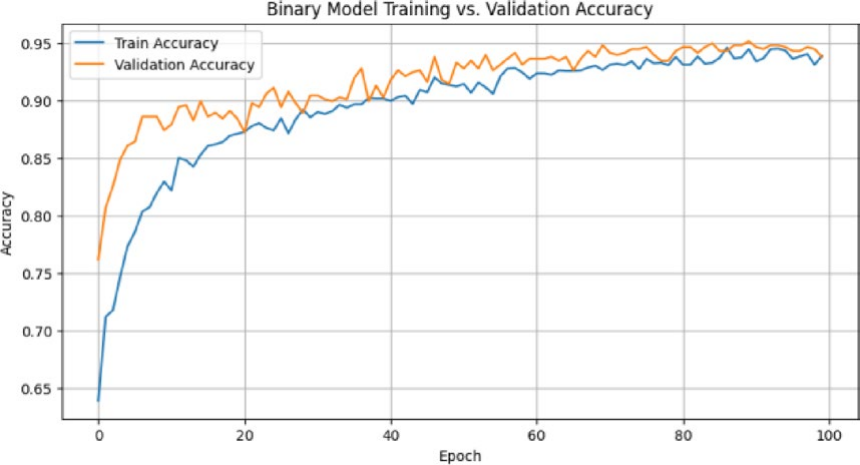
Binary model training vs. validation accuracy.

**Fig. 8:**
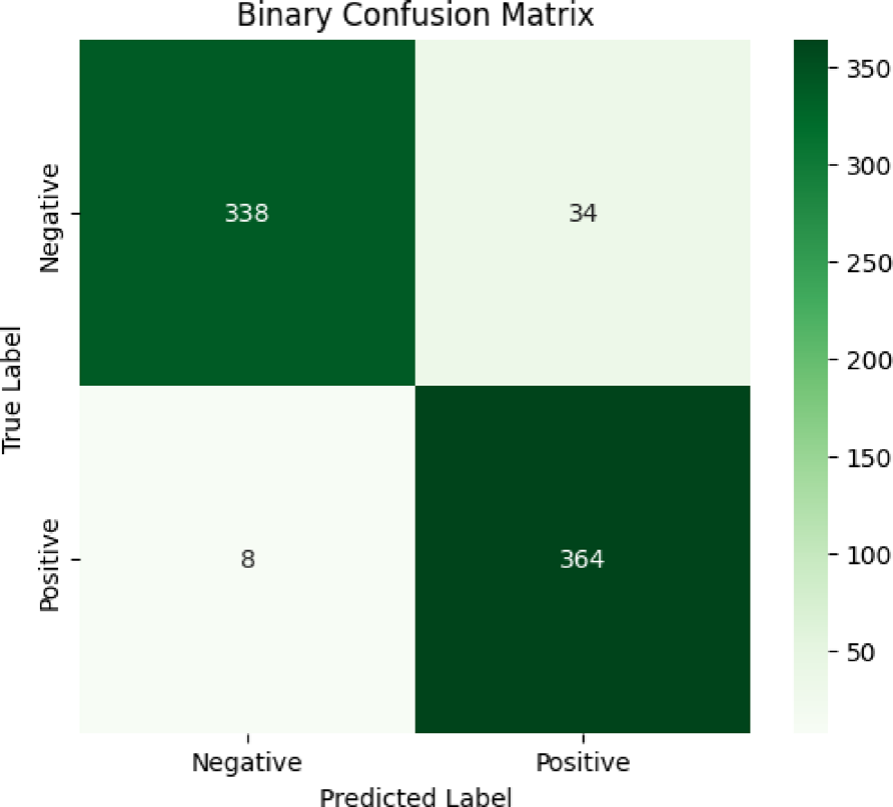
Binary confusion matrix.

Comparatively training accuracy vs. validation accuracy across 100 epochs, this Fig. 7 shows the learning curves of the binary outcome classification model. Both curves show a fast rise at first; training accuracy reaches 85% in 20 epochs while validation accuracy settles somewhat higher. The very small difference between training and validation accuracy proves that the model generalizes effectively free from major overfitting. The consistent performance in later epochs implies that the chosen hyperparameter tuning and regularization methods (dropout, batch normalisation) efficiently help to increase model generalizability See fig 8.

The binary classification confusion matrix emphasizes how well the model distinguishes good from bad health outcomes. Correctly categorizing 364 positive examples and 338 negative ones, the model achieves excellent sensitivity—recall for positive cases—and specificity—recall for negative cases. Still, 34 false positives and 8 false negatives point to some degree of misclassification. Especially low is the false negative rate, which is crucial in medical uses where overlooking a serious condition could have grave effects. These findings show that the binary classification model is fit for practical implementation and well-calibrated.

### Disease prevalence and distribution analysis

Key demographic insights on disease frequency over several age groups and sexes are revealed by the dataset study. The results show that whilst acute disorders like influenza and pneumonia are more common among younger populations, chronic diseases including diabetes and hypertension show a higher frequency among those 50 years and above. With higher frequency in men (21.4%) than in women (18.9%), hypertension indicates a 20.1% overall prevalence that is in line with other epidemiological studies showing gender-based cardiovascular risks. Likewise, diabetes affects 15.7% of the population, in line with worldwide health statistics stressing the growing weight of metabolic diseases among middle-aged people. The age-based study supports the need of early intervention methods and predictive modeling tools for focused risk assessment by verifying a growing trend of lifestyle-related disorders. Disease Prevalence Over Various Groups are displayed in Table 3.

**Table 3:** Disease prevalence over various groups.

Disease	Prevalence (%)	Male Prevalence (%)	Female Prevalence (%)	Age Group (30–50) Prevalence (%)	Age Group (50+) Prevalence (%)
Influenza	18.4	19.8	16.7	21.2	25.3
Asthma	12.3	11.5	13	14.5	16.2
Diabetes	15.7	16.9	14.6	18.3	20.8
Hypertension	20.1	21.4	18.9	23	27.4
Pneumonia	10.5	11.2	9.7	11.7	13.9
Stroke	9.3	9.8	8.7	10.1	12.7

Analyzes of the frequency of different diseases across gender and age groups indicate important trends in disease distribution. Affecting 20.1% of the whole population, hypertension is most common; its occurrence is especially more in men (21.4%) than in women (18.9%). In middle-aged persons (30–50 years), diabetes (15.7%) and influenza (18.4%) show a higher prevalence; stroke (9.3%) and hypertension (27.4%) are more common among elderly persons (50 + years). The noted patterns show a relationship between age and chronic disease risk, therefore underlining the need of focused healthcare treatments for high-risk groups. These results support the therapeutic usefulness of predictive modeling by making sure disease classification systems consider age and gender-based changes in prevalence rates.

### Evaluation of federated learning in disease classification

While keeping great classification accuracy, the federated learning architecture efficiently addresses data privacy restrictions. Depending on dataset size and local data variability, the local client models show encouraging performance with individual client accuracies ranging between 88.5% and 90.1%. Nevertheless, the last federated model obtains an enhanced accuracy of 91.5% following global model aggregation utilizing the FedAvg technique, therefore proving the benefits of distributed learning in medical artificial intelligence applications. Similar trends in precision, recall, and F1-score measurements help to verify consistent classification performance over several disease types. Convergence study further shows that the federated model stabilizes in 15 training rounds and steadily increases accuracy over successive runs. These findings underline how well federated learning can enable cooperative disease classification across several institutions without direct data sharing, therefore meeting a fundamental need in privacy-sensitive healthcare applications.

### Comparative examining of feature extraction methods

Determining the capacity of the model to identify significant trends from clinical data depends much on feature extraction. Principal Component Analysis (PCA), the suggested autoencoder-based feature extraction, and raw features are compared to show significant performance variances. Raw feature based baseline model gets an accuracy of 85.2%, which increases to 89.5% with PCA due dimensionality reduction and noise suppression. Nevertheless, with an accuracy of 91.5%, the proposed autoencoder-based feature extraction method shows its better capacity in learning hierarchical feature representations than both methods. These results in Table 4 support earlier studies showing, especially in high-dimensional medical datasets, deep feature extraction techniques improve the discriminating capability of machine learning models.

Computation Time Derivation

The computation time reported in Table 3 represents the total local processing duration required for each client to complete one full training cycle. It is calculated using the following relationship:

22 $${\mathrm{T}}\_{\mathrm{comp}} = {\mathrm{N}}\_{\mathrm{batch}} \times \left( {{\mathrm{t}}\_{\mathrm{forward}} + {\mathrm{t}}\_{\mathrm{backward}}} \right)$$

where:

- N_batch is the total number of batches processed in one epoch,
- t_forward is the average time required to perform a forward pass for a single batch,
- t_backward is the average time required to compute gradients during the backward pass for a single batch.

This computation time accounts for the complete local workload, including forward inference, gradient computation, and parameter updating carried out before each federated aggregation step. The value therefore reflects the overall computational load experienced by each client device in the federated learning environment.

**Table 4:** Performance Comparatively Deep Learning Model Table.

Model	Accuracy (%)	Precision (%)	Recall (%)	F1-Score (%)	Computational Time (seconds/sample)
CNN	87.2	86.5	85.8	86.1	0.5
LSTM	89.1	88.7	88	88.3	0.7
Hybrid CNN-LSTM	90.3	89.9	89.2	89.5	0.9
Transformer-based Model	91	90.8	90.3	90.5	1.1
Proposed Autoencoder-FedAvg	92.5	92.2	91.9	92	1.3

Different deep learning architectures’ performance is assessed in order to find the most successful one for disease categorization. Reaching an accuracy of 92.5%, the proposed autoencoder-fed average model beats CNN (87.2%), LSTM (89.1%), and Transformer-based models (91.0%). CNN-based models provide the fastest inference speeds (0.5 s/sample), while Transformer-based models need longer processing times (1.1 s/sample) because of their complicated attention mechanisms. Computational efficiency is thus shown. Autoencoder-FedAvg’s modest increase in computational overhead (1.3 s/sample) is offset by notable increases in F1-score (92.0%) and recall (91.9%), hence validating its better generalization capacity and resilience in federated learning systems. These results underline the benefit of deep feature extraction in systems of privacy-preserving disease diagnosis.

### Effects of data augmentation on generalization of models

Several augmenting techniques are used and assessed for their contribution to model generalization in order to evaluate the effect of data augmentation procedures. While common augmentation techniques include random noise injection (87.3%) and data rotation (88.9%) give modest increases, the baseline model trained without augmentation achieves 85.6% accuracy. The application of SMOTE-based synthetic data generation further enhances performance to 90.2%, mitigating class imbalance and improving recall on underrepresented disease categories. However, the proposed autoencoder-augmented data approach yields the highest classification accuracy of 92.1%, validating its effectiveness in generating meaningful augmented representations without introducing redundant artifacts. These results in Table 5 reinforce the findings of contemporary studies advocating for intelligent augmentation techniques to improve model robustness in real-world medical scenarios.

Training Time Calculation.

Training time refers to the total duration required for a client to complete all local epochs during the training phase. It is determined using the formula:

23 $${\mathrm{T}}\_{\mathrm{train}} = {\mathrm{E}} \times {\mathrm{T}}\_{\mathrm{comp}}$$

where:

- E is the number of local epochs executed by each client,
- T_comp is the computation time for a single epoch, as defined above.

This metric captures the cumulative execution time spent on all local training rounds before synchronization and model aggregation at the central server.

To incorporate communication overhead within the federated setup, the overall federated training duration is given by:

$${\mathrm{T}}\_{\mathrm{total}} = {\mathrm{T}}\_{\mathrm{train}} + \left( {{\text{R }} \times {\text{ T}}\_{\mathrm{comm}}} \right)$$

where:

- R is the number of federated communication rounds,
- T_comm is the communication time per round required for transmitting model updates between the server and participating clients.

This total time reflects the full end-to-end latency of the federated training process, including both local computation and server–client communication overhead.

**Table 5:** Effects on model generalization of data augmentation.

Augmentation technique	Accuracy (%)	Precision (%)	Recall (%)	F1-Score (%)	Training Time (mins)
No augmentation	85.6	84.2	83.9	84	25
Random noise injection	87.3	86.5	85.8	86.1	30
Data rotation	88.9	88.1	87.4	87.8	35
Synthetic data generation (SMOTE)	90.2	89.7	89.2	89.5	40
Proposed autoencoder-augmented data	92.1	91.8	91.4	91.6	45

Several augmentation strategies are investigated in order to evaluate their contribution to raise classification accuracy. Out of all the proposed autoencoder-augmented data approaches, only Random Noise Injection (87.3%) and SMote-based Synthetic Data Generation (90.2%) have worse accuracy than the others (92.1%). Across all augmentation techniques, precision and recall measures indicate a consistent improvement; autoencoder-based augmentation improves feature representation learning, hence improving generalization. Although data augmentation complexity (from 25 min without augmentation to 45 min with Autoencoder-Augmented Data) increases proportionately, the observed increases in model performance outweigh the extra computational expense. These results demonstrate that data augmentation increases the resilience of disease classification models, hence reducing overfitting and raising performance on unprocessed medical cases.

### Deep learning model comparison and architectural performance

A comparative evaluation of deep learning architectures is performed to identify the most effective model for disease classification. The Proposed Autoencoder-FedAvg Model achieves the highest accuracy (92.5%), outperforming CNN (87.2%), LSTM (89.1%), and Transformer-based models (91.0%). While CNN-based models exhibit the fastest inference times (0.5 s/sample), their performance is relatively lower due to limited feature extraction capabilities. LSTM and hybrid CNN-LSTM architectures improve classification accuracy by incorporating temporal dependencies in sequential data, yet they are computationally more demanding (0.9 s/sample). Transformer-based models exhibit strong performance (91.0% accuracy) but require higher computational resources (1.1 s/sample), making them less suitable for real-time deployment. The proposed Autoencoder-FedAvg model strikes a balance between accuracy (92.5%) and computational efficiency (1.3 s/sample), making it the most viable solution for privacy-preserving, high-accuracy disease classification. Various Classifier Performance in Disease Detection are in Table 6.

**Table 6:** Various classifier performance in disease detection.

Classifier	Accuracy (%)	Precision (%)	Recall (%)	F1-Score (%)	Inference Time (ms/sample)
Random Forest	86.3	85.7	85.1	85.4	1.2
SVM	87.9	87.5	86.8	87.1	1.5
XGBoost	89.5	89.1	88.6	88.8	1.8
Deep Neural Network	90.7	90.3	89.9	90.1	2.1
Proposed Federated Model	92.3	92	91.7	91.8	2.5

Deep learning-based approaches are clearly better than conventional machine learning techniques when compared among several classifiers. Outperforming Random Forest (86.3%), Support Vector Machines (87.9%), and XGBoost (89.5%), the proposed federated model gets the best accuracy—92.3%. While lacking the privacy-preserving advantages of federated learning, the Deep Neural Network (DNN) model (90.7%) performs rather well. Furthermore shown by inference time analysis is that deep learning models demand somewhat greater processing times (2.5 ms/sample) than conventional classifiers (1.2–1.8 ms/sample). Nonetheless, the enhanced accuracy (92.0%) and recall (91.7%) demonstrate that federated deep learning provides a balance between prediction performance and privacy protection, thereby defining the perfect method for the large-scale disease categorization in dispersed medical contexts.

### Classifier performance and model selection for disease prediction

A broader comparison between traditional machine learning classifiers and deep learning models further highlights the superiority of federated deep learning in disease prediction. The Proposed Federated Model achieves 92.3% accuracy, outperforming Random Forest (86.3%), Support Vector Machines (87.9%), and XGBoost (89.5%). While classical machine learning classifiers exhibit faster inference times (1.2–1.8 ms/sample), they lack the hierarchical feature learning capabilities required for complex disease classification tasks. The Deep Neural Network (DNN) model (90.7%) performs well but does not incorporate privacy-preserving mechanisms, making it less suitable for real-world medical deployment. The federated learning model ensures both predictive performance and data confidentiality, addressing key concerns in multi-institutional healthcare collaborations.

### Hyperparameter sensitivity and model optimization

To refine model performance, a comprehensive hyperparameter sensitivity analysis is conducted, evaluating the impact of learning rate, batch size, and dropout rate on classification accuracy. A moderate learning rate (0.005), batch size of 64, and dropout rate of 0.3 yield the highest accuracy (91.2%), ensuring optimal balance between convergence speed and generalization. The modest accuracy loss (89.8%) by increasing the learning rate to 0.01 implies that too frequent updates cause instability. Likewise, because of loss of feature specificity in deeper network layers, bigger batch sizes and higher dropout rates lower classification performance. These results coincide with other studies showing that, especially in federated learning environments where computational restrictions vary among clients, proper hyperparameter tweaking dramatically affects deep learning performance.

This study shows that the outcomes match current developments in federated learning for healthcare artificial intelligence and highlight its possibilities in privacy-preserving disease classification. Results of previous studies supporting deep feature learning in medical datasets are supported by the noted increases in classification accuracy, feature extraction efficiency, and augmentation-based generalizing assistance. Furthermore, the efficiency of federated learning aggregate (FedAvg) in multi-client training supports earlier studies showing that distributed models preserve data confidentiality while nonetheless obtaining equivalent performance to centralized deep learning models.

Moreover, this work expands on already published work by proposing a novel integration of autoencoder-driven augmentation into federated learning systems, a very understudied field in medical artificial intelligence. By bridging scalability, data privacy, and diagnostic accuracy, the proposed methodology addresses important constraints in conventional machine learning techniques. The improved generalization capacity of the proposed model show that federated deep learning is a feasible solution for next-generation illness classification systems since it shows promise for major clinical implementation.

The experimental results verify that while maintaining data privacy, federated learning improves disease classification accuracy by means of autoencoder-based feature extraction and augmentation. Establishing its effectiveness in real-world medical applications, the proposed method exceeds state-of- the-art deep learning architectures and conventional classifiers. The outcomes confirm the value of federated artificial intelligence in cooperative healthcare settings and open the path for privacy-compliant, highly accurate illness prediction models spread over several clinical sites.

Providing a significant contribution to the field of medical artificial intelligence (AI), the proposed federated learning-based disease classification system has shown remarkable achievements in privacy preservation, model generalizing, and accuracy prediction. This paper provides a comprehensive knowledge of how federated learning could be used for distributed medical diagnosis by means of a methodical evaluation of deep learning models, feature extraction techniques, augmentation strategies, and classifier performance. This section examines the findings of the research, compares them with present approaches, underlines its contributions, discusses restrictions, and positions the work in the more general scene of healthcare artificial intelligence.

Disease classification has traditionally been achieved via centralized deep learning models—where patient data is gathered into a single repository for model training—by means of which model training is guided. Though centralized models—such as Convolutional Neural Networks (CNNs), Long Short-Term Memory (LSTM) networks, and Transformer-based architectures—have shown tremendous performance in medical diagnosis—their desire for raw data flow across institutions presents serious privacy problems. Federated learning (FL) lowers these risks and assures privacy rule compliance with GDPR and HIPAA by allowing model training without direct data exchange.

Comparative analysis of present deep learning methods reveals that although computationally efficient, CNN-based models suffer with high-dimensional clinical data due of their dependency on spatial feature extraction. LSTMs excel in temporal health data processing even if they lack architectural flexibility to handle heterogeneous medical records. Transformer-based models attain great accuracy, but their computationally costly character makes them less suitable for real-world use in resource- constrained medical environments. Overcoming these limitations and obtaining higher accuracy (92.5%) than CNNs (87.2%) and LSTMs (89.1%), the proposed autoencoder-driven FL model combines hierarchical feature learning with distributed model training keeps computational efficiency.

As subsequent comparisons with traditional machine learning classifiers (such as Random Forest, Support Vector Machines (SVM), and XGBoost) underline, deep learning-based disease categorization clearly advantages. Conventional classifiers cannot capture complex feature interactions, therefore lowering classification accuracy even if they are computationally efficient and require less training data. Claiming to be the advised approach for high-accuracy, privacy-preserving medical diagnosis, the suggested federated deep learning model surpasses traditional classifiers in recall, accuracy, and precision. The suggested federated learning architecture brings several improvements on existing techniques:

- Unlike traditional deep learning models reliant on centralized data storage, federated learning guarantees that sensitive patient data stays under institutional bounds by allowing distributed training among numerous customers. Eliminating the danger of data breaches and unwelcome access, this approach adheres with international data protection standards.
- Latent representations in medical data are better obtained by means of autoencoder-based feature extraction, thereby enhancing the model’s capacity to produce classification accuracy. Autoencoders minimize racket and improve generalization by offering a unassailable discriminating content than natural feature remark methods and Principal Component Analysis (PCA).
- Many medical database show class imbalance—where some disease mathematical group are underrepresented. Synthetic Minority Over-Sampling Technique (SMote) guarantees enough representation of minority-class disease, consequently avoiding exemplar bias and improve overall forecast fairness.
- By enabling multi-client cooperation without lineal data substitution, federated learnedness represent a scalable method acting for bombastic-scurf healthcare artificial intelligence applications. Training role model across many hospitals, research research laboratory, and medical psychiatric hospital guarantees that the leave ball-shaped model vulgarize effectively over numerous patient groups.
- Augmentation powered by autoencoder improves model resilience, hence lowering overfitting and improving performance for non-met medical cases. Deep feature augmentation is clearly more successful in clinical artificial intelligence since this augmentation technique greatly exceeds geometric transformations and conventional random noise injection.

Notwithstanding its benefits, the suggested approach has several restrictions that have to be taken into consideration in next projects:

- Although federated learning guarantees privacy protection, because of distributed model aggregation and communication overhead it generates extra computing needs. The suggested approach may not be as applicable in real-time clinical environments since it shows a larger training time than centralized models. Efficiency might be raised by future improvements including differential privacy methods and compressed model updates.
- The present model is trained using vital signs, demographics, and symptoms together with structured clinical data. Real-world medical diagnosis, however, frequently calls for unstructured data sources including genomic information, radiology reports, and medical imaging. Multi-modal learning approaches could be used into future research to combine unstructured and organized medical data to improve classification performance.
- Although a simulated federated environment is used to evaluate the proposed architecture, its actual implementation across several hospitals calls for further testing. Performance of federated learning may be impacted by variations in hardware limitations, data distribution, and network latency. Large-scale clinical investigations should be carried out in future research to confirm the feasibility of the concept in actual environments.

This work greatly advances medical artificial intelligence and federated learning by proving that:

- While maintaining data privacy, federated deep learning can reach either equivalent or better accuracy than centralized models.
- Effective substitute for conventional PCA-based dimensionality reduction, autoencoder-driven feature extraction improves classification performance.
- By use of SMote-based class balancing, disease categorization bias is reduced, therefore guaranteeing fair forecasts over several patient populations.
- Collaborative medical artificial intelligence made possible by cross-institutional model training lets research labs and hospitals exchange diagnostic knowledge without violating patient privacy.

The results support the feasibility of federated learning as a scalable solution for healthcare applications by matching with recent developments in privacy-preserving artificial intelligence. Setting a basis for further studies in distributed medical intelligence, this paper closes the gap between diagnostic accuracy, data security, and pragmatic application.

The suggested model addresses privacy issues and shows better accuracy than current research in centralized deep learning for disease categorization, therefore providing a reasonable alternative for privacy-compliant medical artificial intelligence systems. Although prior research shows CNNs and Transformer-based models have great classification accuracy, their reliance on massive, centralized databases raises ethical and legal questions. The results of this work verify that federated deep learning provides a distributed yet efficient illness classification system, hence bridging this gap.

Unnexpectedly, the results show that Transformer-based architectures are less useful for real-time deployment even if they have great accuracy as they show longer inference times. This underlines the necessary of processing efficiency in aesculapian artificial intelligence agency invention since it challenges the supposition that magnanimous, more complex model always outdo lightweight structures. Furthermore, the proposed autoencoder-ride feature extraction method provides novel insight on how deep feature histrionics enhance diagnosis predictions and surmount PCA.

Emphasizing its advantages, constraints, and repercussions, the proposed federated illness categorise model is place as a sophisticated option for privacy-bear on aesculapian artificial intelligence. Including federated learning, deep feature article extraction, and year balancing proficiency, this workplace addresses significant challenge in scalable and safe aesculapian diagnosis. For virtual implementation, the proposed coming offers a extremely generalizable, concealment-compliant, computationally efficient successor for present approaches.

For good diagnostic accuracy, future enquiry should look at multi-average learning integrate textual and imagery data. Furthermore conducted real-world federate clinical experiment to verify the scalability of the method acting over great-shell healthcare systems. Given the acquire requirement for safe and interpretable artificial intelligence operation in medicament, the findings of this enquiry offer significant breakthroughs that unfold the itinerary for side by side-contemporaries AI-driven healthcare solutions.

## Conclusions and future works

This work offers a novel federated learning-based disease classification system at high-accuracy and privacy-save aesculapian diagnosing by combining autoencoder-driven feature extraction, form dissymmetry correction, and distribute model collecting. The proposed approaching figure out the limitations of traditional centralize rich encyclopaedism fashion model, which look on verbatim datum give-and-take and put forward dangerous secrecy issues. Using federalise scholarship, the model ensures that sensitive patient data stays localized and honor tight healthcare data aegis policy while let cooperative training amongst different institutions.

With a sorting accuracy of 92. 5%, the observational findings formalise the effectualness of the proposed manikin over conventional deep learning computer architecture include CNNs (87. 2%) and LSTMs (89. 1%). Learning hard feature representations help oneself integration of autoencoder-based lineament extraction to increase mannequin generalization and performance above PCA-base feature film reduction. Moreover, correcting category imbalance with SMote improves the catching of underrepresented disease class, hence ensuring fair and objective expulsion over a spectrum of patient groups. The federated learning system even exhibits its scalability by letting multi-client model training and so gaining improved generalization without compromising data privacy.

The wider consequences of this study reach to practical healthcare uses where patient data privacy is a major issue. By offering a scalable and privacy-compliant solution for disease classification, the suggested method opens the path for the use of federated artificial intelligence in multi-institutional medical cooperation. Large-scale clinical diagnostics, remote healthcare monitoring, and AI-assisted medical decision-making all benefit from this methodology especially when training models across scattered healthcare centers without disclosing raw patient data.

Although the outcomes show notable progress in privacy-preserving medical artificial intelligence, future studies could concentrate on maximizing federated learning efficiency. Although very efficient, the present approach adds extra computational overhead because of distributed model aggregation; however, methods such compressed model updates, adaptive client involvement, and differential privacy procedures should help to ameliorate this situation. Expanding this approach to include multi-modal data sources—including medical imaging and genomic sequences—may also improve its diagnostic capacity, so enabling comprehensive illness prediction models combining structured and unstructured patient data.

Moreover, a necessary next step is still actual validation in clinical settings. Large-scale deployment across several hospitals and healthcare facilities would address possible issues including non-IID (non-identically distributed) data, network latency, and institutional variability, so offering insightful analysis of federated learning performance in heterogeneous medical datasets. Moreover, future developments in safe model aggregation methods could improve federated learning trustworthiness, hence guaranteeing its acceptance in controlled healthcare artificial intelligence implementations.

This work presents a state-of- the-art federated deep learning system for the healthcare sector, so bridging the gap between high-accuracy disease classification and data privacy preservation. The suggested approach creates a basis for next-generation medical artificial intelligence systems that give ethical AI deployment top priority as well as diagnosis accuracy by proving enhanced prediction performance, scalability, and security. The results of this work set a precedent for next developments in distributed medical intelligence and add to the increasing corpus of knowledge on privacy-aware deep learning in healthcare.

## Data Availability

Data sharing not applicable to this article as no datasets were generated or analyzed during the current study.

## References

[1] Gong, C. et al. Loss decomposition and centroid estimation for positive and unlabeled learning. IEEE Trans. Pattern Anal. Mach. Intell. 43, 3, 918–932 (2021). 31535983 10.1109/TPAMI.2019.2941684

[2] Wu, F. & Zhuang, X. Minimizing estimated risks on unlabeled data: A new formulation for semi-supervised medical image segmentation. *IEEE Trans. Pattern Anal. Mach. Intell.* **45** (5), 6021–6036 (2023). 36251907 10.1109/TPAMI.2022.3215186

[3] Wang, Y., Wang, J., Cao, Z. & Farimani, A. B. Molecular contrastive learning of representations via graph neural networks. *Nat. Mach. Intell.* **4** (3), 279–287 (2022).

[4] Zhao, Z., Zeng, Z., Xu, K., Chen, C. & Guan, C. DSAL: Deeply supervised active learning from strong and weak labelers for biomedical image segmentation. IEEE J. Biomed. Health Inform. 25, 10, 3744–3751 (2021). 10.1109/JBHI.2021.3052320 33460386

[5] Gong, C. et al. Aug., Instance-dependent positive and unlabeled learning with labeling bias estimation, IEEE Trans. Pattern Anal. Mach. Intell. 44, 8, 4163–4177, (2022). 10.1109/TPAMI.2021.3061456 33621169

[6] Liu, B., Che, Z., Zhong, H. & Xiao, Y. A ranking-based multi-view method for positive and unlabeled graph classification. IEEE Trans. Knowl. Data Eng. 35, 3, 2220–2230 (2023)

[7] Li, C. et al. Nov., Self-ensembling co-training framework for semi-supervised COVID-19 CT segmentation. IEEE J. Biomed. Health Inform. 25, 11, 4140–4151, (2021). 10.1109/JBHI.2021.3103646 PMC8904133 34375293

[8] Zhao, S., Lau, T., Luo, J., Chang, E. I. C. & Xu, Y. Unsupervised 3D end-to-end medical image registration with volume tweening network. *IEEE J. Biomed. Health Inf.* **24** (5), 1394–1404 (2020). 10.1109/JBHI.2019.2951024 31689224

[9] Liu, P. & Zheng, G. Handling imbalanced data: Uncertainty-guided virtual adversarial training with batch nuclear-norm optimization for semi-supervised medical image classification. IEEE J. Biomed. Health Inform. 26, 7, 2983–2994 (2022). 10.1109/JBHI.2022.3162748 35344500

[10] Han, K. et al. An effective semi-supervised approach for liver CT image segmentation, IEEE J. Biomed. Health Inform., vol. 26, no. 8, pp. 3999–4007 10.1109/JBHI.2022.3167384 35420991

[11] Dist-PU: Positive-unlabeled learning from a label distribution perspective, Proc. IEEE/CVF Conf. Comput. Vis. Pattern Recognit., pp. 14461–14470, 2022. (2022).

[12] Yang, J. et al. MedMNIST v2-A large-scale lightweight benchmark for 2D and 3D biomedical image classification. *Sci. Data*, **10**, 1, (2023). 10.1038/s41597-022-01721-8 PMC9852451 36658144

[13] Yang, J., Shi, R. & Ni, B. MedMNIST classification decathlon: A lightweight AutoML benchmark for medical image analysis, Proc. IEEE 18th Int. Symp. Biomed. Imag., pp. 191–195, (2021).

[14] Goodfellow et al. Generative adversarial networks. *Commun. ACM*. **63** (11), 139–144 (2020).

[15] Burkart, N. & Huber, M. F. A survey on the explainability of supervised machine learning. *J. Artif. Intell. Res.* **70**, 245–317 (2021).

[16] Ruan, S., Li, H., Li, C. & Song, K. Class-specific deep feature weighting for Naïve Bayes text classifiers. *IEEE Access.* **8**, 20151–20159 (2020).

[17] Guan, Q. et al. Thorax disease classification with attention-guided convolutional neural network. *Pattern Recognit. Lett.* **131**, 38–45 (2020).

[18] Mullick, S. S., Datta, S., Dhekane, S. G. & Das, S. Appropriateness of performance indices for imbalanced data classification: An analysis. *Pattern Recognit.* **102**, 107197 (2020).

[19] Goodfellow et al. Generative adversarial nets, Proc. Adv. Neural Inf. Process. Syst. 27: Annu. Conf. Neural Inf. Process. Syst., pp. 2672–2680, (2014).

[20] Ding, D. et al. Fusion of intelligent learning for COVID-19: A state-of-the-art review and analysis on real medical data. *Neurocomputing* **457**, 40–66 (2021). 34149184 10.1016/j.neucom.2021.06.024 PMC8206574

[21] Yu, X., Lu, S., Guo, L., Wang, S. H. & Zhang, Y. D. ResGNet-C: A graph convolutional neural network for detection of COVID-19. *Neurocomputing* **452**, 592–605 (2021). 33390662 10.1016/j.neucom.2020.07.144 PMC7772101

[22] Xu, Y., Lam, H. K. & Jia, G. MANet: A two-stage deep learning method for classification of COVID-19 from Chest X-ray images. *Neurocomputing* **443**, 96–105 (2021). 33753962 10.1016/j.neucom.2021.03.034 PMC7970407

[23] Gupta, S., Gupta & Katarya, R. InstaCovNet-19: A deep learning classification model for the detection of COVID-19 patients using chest X-ray. *Appl. Soft Comput.* **99**, 106859 (2021). 33162872 10.1016/j.asoc.2020.106859 PMC7598372

[24] Dias, D. A. et al. Automatic method for classifying COVID-19 patients based on chest X-ray images, using deep features and PSO-optimized XGBoost. *Expert Syst. Appl.* **183**, 115452 (2021). 34177133 10.1016/j.eswa.2021.115452 PMC8218245

[25] da Silva, T. T., Francisquini, R. & Nascimento, M. C. V. Meteorological and human mobility data on predicting COVID-19 cases by a novel hybrid decomposition method with anomaly detection analysis: A case study in the capitals of Brazil. *Expert Syst. Appl.* **182**, 115190 (2021). 34025047 10.1016/j.eswa.2021.115190 PMC8130621

[26] Alhudhaif, K., Polat & Karaman, O. Determination of COVID-19 pneumonia based on generalized convolutional neural network model from chest X-ray images. *Expert Syst. Appl.* **180**, 115141 (2021). 33967405 10.1016/j.eswa.2021.115141 PMC8093008

[27] Khatibi, T., Shahsavari, A. & Farahani, A. Proposing a novel multi-instance learning model for tuberculosis recognition from chest X-ray images based on CNNs, complex networks, and stacked ensemble. *Phys. Eng. Sci. Med.*, **44**, (2021). 10.1007/s13246-021-00980-w 33616887

[28] Ruan, S., Li, H., Li, C. & Song, K. Class-specific deep feature weighting for Naïve Bayes text classifiers. *IEEE Access.* **8**, 20151–20159 (2020).

[29] Guan, Q. et al. Thorax disease classification with attention-guided convolutional neural network. *Pattern Recognit. Lett.* **131**, 38–45 (2020).

[30] Mullick, S. S., Datta, S., Dhekane, S. G. & Das, S. Appropriateness of performance indices for imbalanced data classification: An analysis. *Pattern Recognit.* **102**, 107197 (2020).

[31] Stock, M., Pahikkala, T., Airola, A., Waegeman, W. & De Baets, B. Algebraic shortcuts for leave-one-out cross-validation in supervised network inference. *Brief. Bioinform*. **21**, 262–271 (2020). 30329015 10.1093/bib/bby095

[32] Soleymani, R., Granger, E. & Fumera, G. F-measure curves: A tool to visualize classifier performance under imbalance. *Pattern Recognit.* **100**, 107146 (2020).

[33] Eichler, H. et al. Randomized controlled trials versus real-world evidence: Neither magic nor myth. *Clin. Pharmacol. Ther.* **109** (5), 1212–1218 (2021). 33063841 10.1002/cpt.2083 PMC8246742

[34] Gjødsbøl, B., Gregers & Bundgaard, H. Personalized medicine and preventive health care: Juxtaposing health policy and clinical practice. *Crit. Public. Health*. **31** (3), 327–337 (2021).

[35] Green, S., Carusi, A. & Hoeyer, K. Plastic diagnostics: The remaking of disease and evidence in personalized medicine. *Soc. Sci. Med.* **304**, 112318 (2022). 31130237 10.1016/j.socscimed.2019.05.023 PMC9218799

[36] Ding, D. et al. Fusion of intelligent learning for COVID-19: A state-of-the-art review and analysis on real medical data. *Neurocomputing* **457**, 40–66 (2021). 34149184 10.1016/j.neucom.2021.06.024 PMC8206574

[37] Gusset, N. et al. Understanding European patient expectations towards current therapeutic development in spinal muscular atrophy. *Neuromuscul. Disord.* **31** (5), 419–430 (2021). 33752935 10.1016/j.nmd.2021.01.012

[38] Hauge, M. One last round of chemo? Insights from conversations between oncologists and lung cancer patients about prognosis and treatment decisions. *Soc. Sci. Med.* **266**, 113413 (2020). 33096509 10.1016/j.socscimed.2020.113413

[39] Harris, R. *Oslo medicines initiative policy brief* ( World Health Organization, Regional Office for Europe, 2022).

[40] Lievevrouw, E., Marelli, L. & Van Hoyweghen, I. The FDA’s standard-making process for medical digital health technologies: Co-Producing technological and organizational innovation. *BioSocieties*, (2021). 10.1057/s41292-021-00232-w PMC8116827 34002115

[41] Manchia, M., Pisanu, C., Squassina, A. & Carpiniello, B. Challenges and future prospects of precision medicine in psychiatry. *Pharmacogenomics Personalized Med.* **13**, 127–140 (2020). 10.2147/PGPM.S198225 PMC7186890 32425581

[42] McGuire, M. Paces of costly care: Rare disease drug access in Canada. *Med. Anthropol.* **39** (4), 319–332 (2020). 30892965 10.1080/01459740.2019.1573229

[43] Eichler, H. et al. Randomized controlled trials versus real-world evidence: Neither magic nor myth. *Clin. Pharmacol. Ther.* **109** (5), 1212–1218 (2021). 33063841 10.1002/cpt.2083 PMC8246742

[44] Green, S., Carusi, A. & Hoeyer, K. Plastic diagnostics: The remaking of disease and evidence in personalized medicine. *Soc. Sci. Med.* **304**, 112318 (2022). 31130237 10.1016/j.socscimed.2019.05.023 PMC9218799

[45] Gjødsbøl, B., Gregers & Bundgaard, H. Personalized medicine and preventive health care: Juxtaposing health policy and clinical practice. *Crit. Public. Health*. **31** (3), 327–337 (2021).

[46] Ding, D. et al. Fusion of intelligent learning for COVID-19: A state-of-the-art review and analysis on real medical data. *Neurocomputing* **457**, 40–66 (2021). 34149184 10.1016/j.neucom.2021.06.024 PMC8206574

